# Development and validation of a predictive model for cognitive impairment after first-episode acute ischemic stroke without reperfusion therapy

**DOI:** 10.3389/fneur.2026.1731060

**Published:** 2026-03-06

**Authors:** Jiangnan Li, Yufan Pu, Yaran Li, Xuejing Li

**Affiliations:** The Affiliated Huai’an Hospital of Xuzhou Medical University, Huai’an, Jiangsu, China

**Keywords:** acute ischemic stroke, cognitive impairment, interpretability, machine learning, nomogram, predictive model

## Abstract

**Objective:**

To construct and validate a predictive model of cognitive impairment after first-episode acute ischemic stroke (AIS) based on multiple factors.

**Methods:**

A total of 627 patients with first-episode AIS admitted to the Affiliated Huai’an Hospital of Xuzhou Medical University between January 2023 and June 2024 were enrolled in this study. Patients were followed up 6 months after discharge. Cognitive function was assessed using the Mini-Mental State Examination (MMSE). Among them, 188 patients with cognitive impairment were assigned to the cognitively impaired group, and 439 cognitively normal patients were assigned to the non-impaired group. These 627 patients were randomly split 8:2 into a training cohort (*n* = 502) and an internal validation cohort (*n* = 125). Additionally, 184 patients from the Huai’an First People’s Hospital served as the external validation cohort. The least absolute shrinkage and selection operator (LASSO) regression was used to screen for variables significantly associated with cognitive impairment, and multivariate logistic regression was used to further verify their independence. Python and Dataiku DSS platforms were used to build seven machine learning models, including gradient boosted trees, random forest, support vector machine and so on, and the prediction performance of the model was evaluated by AUC value of ROC.

**Results:**

LASSO regression finally identified 10 predictors, such as education level, C-reactive protein (CRP), frontal lobe infarction, number of infarcts. Subsequent multivariable logistic regression analysis revealed that the number of infarcts (OR = 2.17, 95% CI: 1.65–2.86, *p* < 0.001), C-reactive protein level (OR = 1.02, 95% CI: 1.01–1.03, *p* = 0.002), and left-sided frontal lobe infarction (OR = 4.56, 95% CI: 1.97–10.55, *p* < 0.001), among others, were independent risk factors for cognitive impairment after acute ischemic stroke (AIS). In contrast, a higher educational level was identified as a protective factor. Among the machine learning models evaluated, the logistic regression model demonstrated the best performance. It achieved an area under the curve (AUC) of 0.925 (accuracy: 0.871) in the internal validation and an AUC of 0.897 (accuracy: 0.817) in the external validation. The model achieved high predictive accuracy and demonstrated excellent discrimination.

**Conclusion:**

This study successfully constructed a predictive model of cognitive impairment after AIS based on multi-factor analysis and machine learning. By visualizing the model in the form of the nomogram, clinicians can quickly assess the risk based on the individual patient data, thus enabling early identification and individualized intervention.

## 1 Introduction

With the increasing aging of society and the influence of many factors such as unhealthy lifestyle, stroke has become a worldwide problem. Acute ischemic stroke (AIS) is the most common type of stroke. It has the characteristics of high morbidity, high disability rate and high mortality (1). In recent years, the incidence of AIS continues to rise worldwide, especially in China, where AIS accounts for 69.6 to 72.8% of new stroke cases (2, 3). The 2019 Global Burden of Disease Study published in The Lancet Public Health in 2021 showed that the mortality rate of stroke in China ranks first (3). AIS has become one of the most important causes of adult death and long-term disability, which has brought great burden to families and society.

Studies have shown that about 1/3 of stroke patients will have different degrees of cognitive impairment (CI) (4, 5). The cognitive ability of these patients is affected in many aspects such as memory, attention, language and executive function (6–8). Cognitive impairment after AIS not only significantly reduces the quality of life of patients, but also increases the demand for care and economic burden (9, 10). Therefore, the early prediction of cognitive impairment after AIS has become an important research topic in the field of neuroscience.

At present, the prediction model of cognitive impairment after AIS is gradually developing. However, due to the limitations of the accuracy, applicability and extrapolation performance of the existing models, its clinical application still needs to be further improved and verified. By integrating a variety of clinical variables, such as patient’s age, gender, underlying disease, location of cerebral infarction, and levels of inflammatory markers. Machine learning models can be constructed to predict the risk of cognitive impairment in patients with AIS (11–14). Nomogram, as a common prediction tool (15, 16), can quantify these risk factors to help doctors intuitively assess the possibility of cognitive impairment in patients and provide a basis for early intervention.

## 2 Objects and methods

### 2.1 Objects of study

A prospective cohort study design was used in this study. A total of 627 patients with first-episode acute ischemic stroke hospitalized in the Affiliated Huaian Hospital of Xuzhou Medical University from January 2023 to June 2024 were selected. The patients were randomly split into a development cohort (*n* = 502) and an internal validation cohort (*n* = 125) in an 8:2 ratio; 184 patients from Huai’an First People’s Hospital served as an external validation cohort. Inclusion criteria: (1) In accordance with the diagnostic criteria for acute ischemic stroke in the *Guidelines for the Diagnosis and Treatment of Acute Ischemic Stroke in China 2023*, confirmed by MRI with diffusion-weighted imaging (DWI) performed within 72 h of admission; (2) First-ever stroke with symptom onset within 14 days; (3) Age ≥18 years; (4) Able to complete follow-up questionnaires, physical examinations, head MRI and other medical examinations; (5) Participate in the follow-up investigation voluntarily and sign a written informed consent. Exclusion criteria: (1) Patients receiving intravenous thrombolysis, endovascular intervention and bridging therapy within the acute phase time window; (2) Patients who had been diagnosed with dementia before stroke; (3) History of cerebral hemorrhage or subarachnoid hemorrhage, brain trauma, brain tumor, etc.; (4) History of pregnancy, malignant tumors, hematological diseases and infectious diseases; (5) Patients with previous major organ dysfunction or life expectancy of less than 3 years; (6) Patients with congenital mental retardation or other severe neurological and psychiatric diseases in the past; (7) Patients with severe visual or hearing impairment. Written informed consent was obtained from all participants or their legal surrogates. The study protocol was approved by the Medical Ethics Committee of the Affiliated Huai’an Hospital of Xuzhou Medical University.

The sample size was determined by *a priori* power analysis. We aimed to develop a prediction model for cognitive impairment after first-episode AIS, with the occurrence of cognitive impairment (yes/no) as the primary outcome. Based on prior literature, we assumed an odds ratio (OR) of approximately 5.0 for the key imaging predictor “left frontal lobe infarction” (expected proportion in the non-impaired group ~4%). With a two-sided *α* of 0.05 and 80% power, the calculation using G*Power software yielded a minimum required sample size of 371. Accounting for approximately 10% data missingness or attrition, we planned to enroll at least 413 participants. The present study ultimately included 627 participants with 188 cognitive impairment events, which exceeds the pre-calculated requirement and ensures stable model estimation.

### 2.2 Data collection

All patients underwent standardized history and neurological examinations by neurologists. Cognitive assessment was performed at 6 months post-discharge using the MMSE. Based on the 2021 Chinese expert consensus on the management of post-stroke cognitive impairment, cognitive impairment was diagnosed using education-adjusted cut-offs of ≤17 for illiteracy, ≤20 for primary school, and ≤24 for junior high school and above.

Basic data of patients were collected, including gender, age, education level, smoking history, drinking history, hypertension, diabetes, history of coronary artery disease, stroke history, baseline systolic blood pressure and diastolic blood pressure at admission, as well as NIHSS score and ASPECT score. All patients had their elbow venous blood drawn in the morning for routine tests within 24 h after admission and after fasting for more than 8 h, including high-sensitivity C-reactive protein, C-reactive protein, fasting blood glucose, total cholesterol, triglyceride, high-density lipoprotein, low-density lipoprotein, neutrophils, lymphocytes, etc. Based on the cranial MRI examination performed after the onset of the patient’s disease, determine the infarction site, the number of infarcts, degree of cerebral artery stenosis, cerebral artery perfusion, white matter degeneration, etc. in patients.

Criteria for infarct count: The number of acute infarct foci appearing hyperintense on DWI sequences in the acute phase was counted. Isolated, tiny subcortical punctate infarcts (typically with a diameter <3 mm) were excluded. Confluent infarcts within the same vascular territory were counted as a single infarct.

### 2.3 Statistical analysis

In this study, Python, R, SPSS and other statistical software were used for data analysis and processing, and Origin was used for image processing. The normality of measurement data was assessed using the Shapiro–Wilk test, and homogeneity of variance was evaluated using Levene’s test. Data in normal distribution were expressed as mean ± standard deviation, and two independent samples *t*-test was used for comparison between groups. The data that did not conform to normal distribution were described by median and quartile. Nonparametric rank sum test (Mann–Whitney *U* test) was used for comparison between groups. Enumeration data were expressed as cases and percentages (*n*, %), and chi-square test was used for comparison between groups. Variance analysis or non-parametric test (Kruskal–Wallis test) was used to measure the differences of multiple groups of data. Enumeration data were determined by the chi-square test for contingency tables or Fisher’s exact test. Through LASSO regression for variable selection and the Dataiku DSS Visual platform, seven binary machine learning models were built on the development cohort for preliminary comparison. After internal and external validation, the optimal model was chosen for interpretability analysis of individual predictors and for the construction of a clinical nomogram to estimate cognitive impairment risk. The difference was considered statistically significant when *p* < 0.05.

### 2.4 Missing data and handling

A small amount of laboratory data was missing in this study (with ≤5 missing values per individual variable). We handled the missing values using multiple imputation by chained equations (MICE). The imputation was performed in R software (version 4.3.1) using the mice package, applying the predictive mean matching (PMM) model for continuous variables. The imputation model incorporated all available variables in the dataset, including demographic characteristics, clinical variables, imaging indicators, and the outcome variable. We generated *m* = 5 imputed datasets with 10 iterations. Subsequent predictive model building was conducted independently on each imputed dataset, and the final parameter estimates were pooled according to Rubin’s rules.

## 3 Results

### 3.1 Comparison of general clinical data of the participants

A total of 627 patients with first-episode acute ischemic stroke were ultimately enrolled in this study. The patients aged from 29 to 102 years, with an average age of 66.63 ± 11.75 years. Among them, 408 (65.07%) were male and 219 (34.93%) were female. There were 125 patients (19.94%) with a history of smoking, 106 (17.91%) with a history of alcohol consumption, 504 (80.38%) with hypertension, 252 (40.19%) with diabetes mellitus, and 99 (15.79%) with a history of coronary heart disease. At 6 months after acute stroke, 188 patients (29.98%) developed post-stroke cognitive impairment and were designated as the cognitively impaired group; the remaining 439 patients (70.02%) with normal cognition served as the non-impaired group. In the cognitively impaired group, 121 patients (64.36%) were male and 67 (35.64%) were female, with an average age of 69.75 ± 12.16 years. In the non-impaired group, 287 patients (65.38%) were male and 152 (34.62%) were female, with an average age of 65.30 ± 11.33 years (see Supplementary Tables 1, 2).

### 3.2 Screening variables based on LASSO regression to determine the best variables for the prediction model

**Figure 1:**
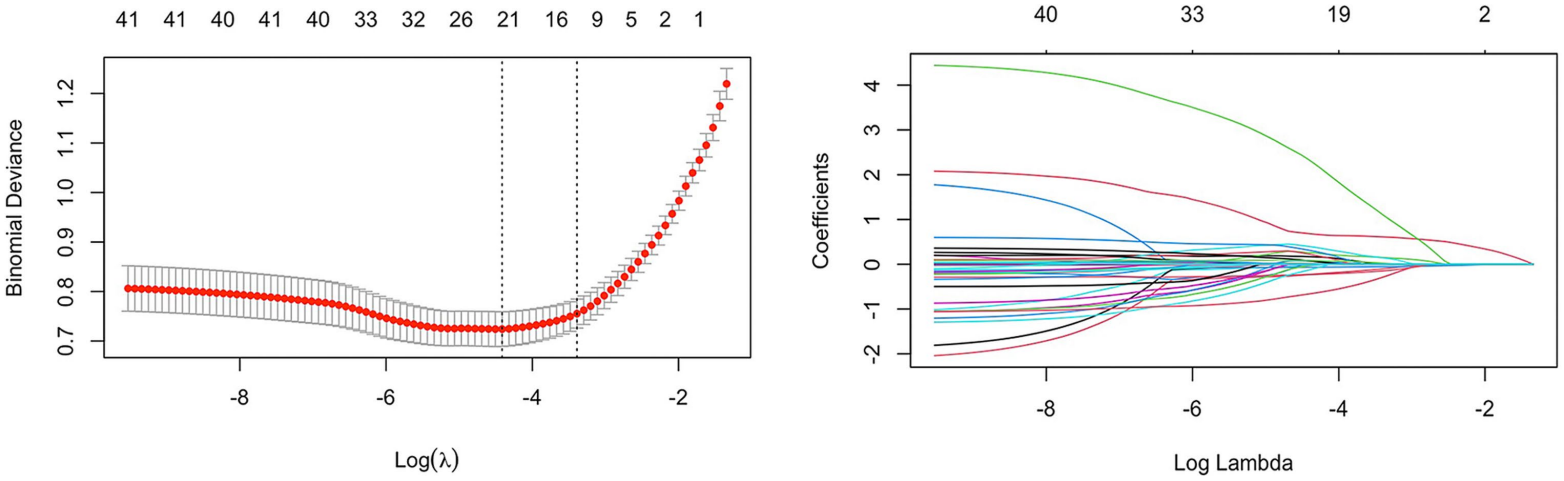
LASSO coefficient curve of cognitive impairment after acute ischemic stroke. Each curve in the right figure shows the change of the coefficient of each variable, the ordinate is the coefficient value, and the lower abscissa is log (*λ*). The upper abscissa is the number of non-zero coefficients in the model at this time. The left panel is a 10-fold cross validation fit followed by a selection of variables.

Predictors of cognitive impairment after acute ischemic stroke were initially screened by LASSO regression only on the training dataset. Variables were centered and normalized by 10-fold cross-validation. The final selected predictors included low density lipoprotein, neutrophil, lymphocytes, C-reactive protein, education level, frontal lobe infarction, temporal lobe infarction, corpus callosum infarction, number of infarction, and degree of cerebral artery stenosis (Figure 1).

### 3.3 Construction and validation of multiple machine learning models

In the development cohort, prediction models were constructed using seven machine learning algorithms. The hyperparameters for each model were optimized via 5-fold cross-validation, with the area under the curve (AUC) serving as the evaluation metric. Using the optimal hyperparameters identified from this tuning process (see Supplementary Table 3), each algorithm was retrained on the full training set. The performance of the models was then evaluated on the internal validation cohort. Logistic regression demonstrated superior performance, achieving an AUC of 0.925 (see Table 1).

**Table 1:** Multi-model internal validation evaluation table.

Model	Accuracy	Precision	Recall	*F*_1_-score	AUC (95% CI)
Logistic regression	0.871	0.768	0.827	0.794	0.925 (0.869–0.981)
SVM	0.864	0.795	0.747	0.767	0.917 (0.856–0.978)
Random forest	0.877	0.843	0.729	0.780	0.915 (0.852–0.978)
XGBoost	0.858	0.772	0.768	0.766	0.910 (0.833–0.987)
Gradient boosted trees	0.869	0.779	0.796	0.785	0.909 (0.862–0.956)
LightGBM	0.861	0.778	0.755	0.763	0.899 (0.839–0.959)
Decision tree	0.828	0.704	0.735	0.717	0.814 (0.775–0.853)

**Figure 2:**
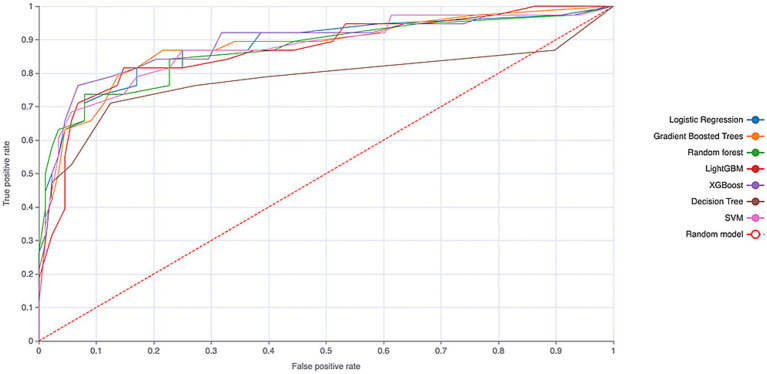
ROC curves of the machine learning models on the external validation cohort.

**Figure 3:**
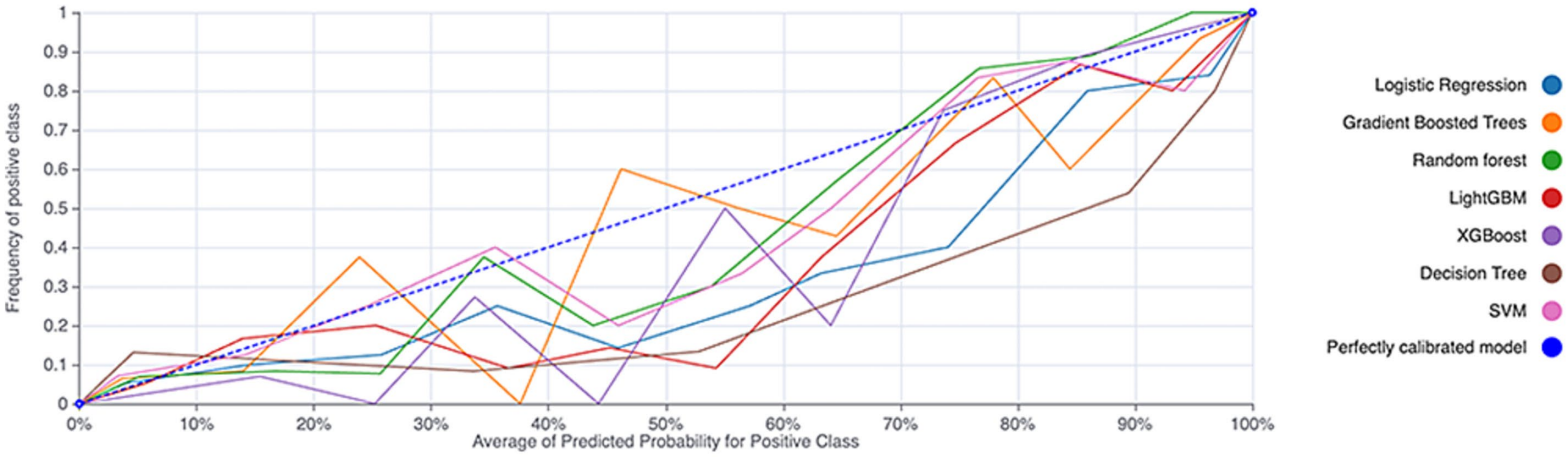
Calibration curves of the machine learning models on the external validation cohort.

During the external validation phase, the models were assessed on an independent dataset to evaluate their generalizability. Logistic regression again performed best, with an AUC of 0.897 (see Figure 2 and Table 2). The calibration curve indicated that the logistic regression model maintained good calibration in the external validation set (see Figure 3), supporting its robust predictive performance.

**Table 2:** Multi-model external validation evaluation table.

Model	Accuracy	Precision	Recall	*F*_1_-score	AUC (95% CI)
Logistic regression	0.817	0.766	0.878	0.812	0.897 (0.836–0.958)
SVM	0.788	0.727	0.880	0.789	0.894 (0.849–0.939)
Random forest	0.830	0.842	0.785	0.807	0.893 (0.824–0.962)
Gradient boosted trees	0.807	0.746	0.886	0.805	0.887 (0.821–0.953)
XGBoost	0.818	0.819	0.785	0.794	0.871 (0.812–0.930)
LightGBM	0.790	0.762	0.818	0.778	0.851 (0.768–0.934)
Decision tree	0.780	0.788	0.741	0.751	0.778 (0.624–0.932)

### 3.4 Interpretable results of logistic regression algorithm model

**Figure 4:**
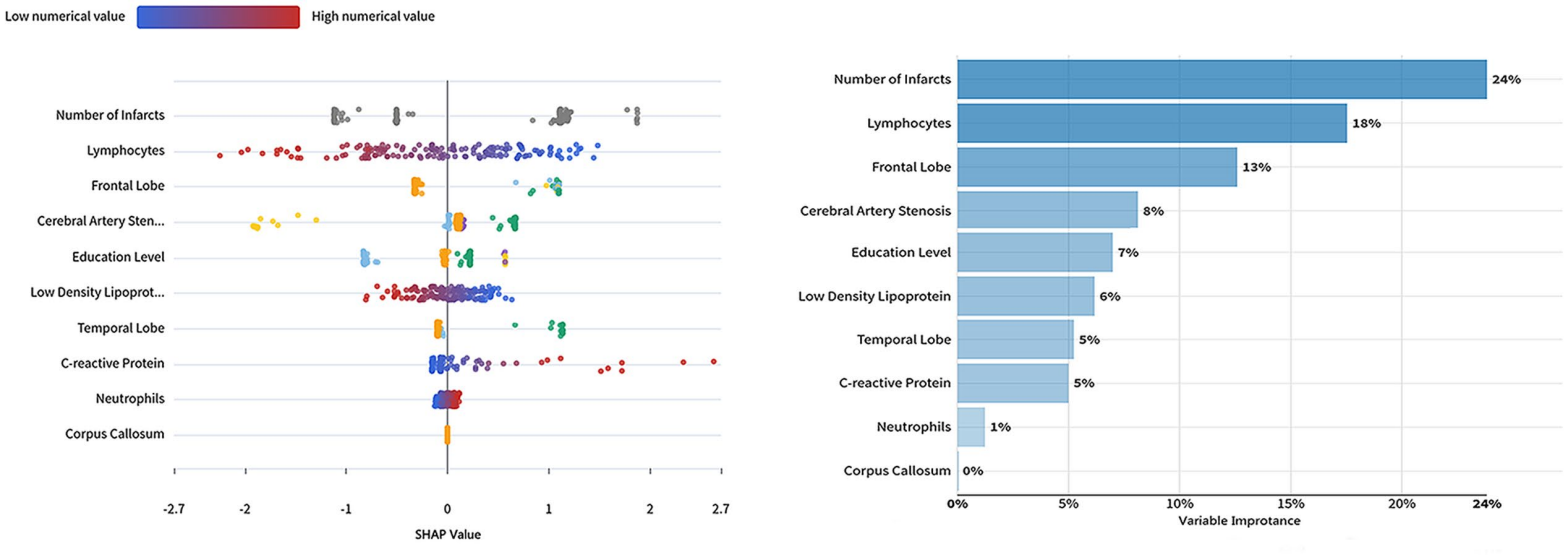
Univariate interpretation and variable importance based on the logistic regression model.

Variable importance ranking based on the logistic regression model is presented. The number of infarcts, lymphocyte ratio, and frontal lobe infarction were the most important variables influencing the prediction, with importance scores of 24, 18, and 13%, respectively. The degree of cerebral artery stenosis had an importance of 8%, followed by education level (7%) and low-density lipoprotein (6%). The importance scores for temporal lobe infarction and C-reactive protein were both 5%. The least important variables were neutrophil ratio and corpus callosum infarction, at 1 and 0%, respectively (see Figure 4).

The left side of Figure 4 displays the individual factor interpretation based on the logistic regression model. The impact of each variable is represented by color and position, with a gradient from blue to red indicating low to high values. Each dot in the plot represents a single sample. A position further to the right denotes a greater positive contribution to the model’s prediction. For instance, the number of infarcts is a key variable; samples with higher values are predominantly clustered in the positive effect region, indicating that a greater number of infarcts is associated with an increased prediction. Furthermore, the variables exhibit different ranges and distribution biases in the plot, reflecting their distinct weights and effects within the model.

Supplementary Figure 1 shows the effect of different factors on the risk of cognitive impairment after stroke.

### 3.5 Construction of a prediction model using multiple logistic regression

We included 10 predictive factors obtained from LASSO regression screening as independent risk factors, following the exclusion of lymphocytes due to multicollinearity concerns (see Supplementary Table 4), we constructed a multivariable logistic regression model (Table 3).

**Table 3:** Results of multivariate logistic regression.

Variables	*β*	SE	*Z*	*p*	OR (95% CI)
Intercept	−1.84	1.75	−1.05	0.293	0.16 (0.01–4.90)
Frontal lobe infarction					
None					1.00 (Reference)
Left side	1.52	0.43	3.54	<0.001	4.56 (1.97–10.55)
Right side	0.78	0.44	1.75	0.080	2.18 (0.91–5.20)
Temporal lobe infarction					
None					1.00 (Reference)
Left side	1.38	0.55	2.53	0.012	3.98 (1.36–11.61)
Right side	−0.01	0.60	−0.01	0.993	0.99 (0.30–3.25)
Corpus callosum infarction					
None					1.00 (Reference)
Have	1.95	0.86	2.28	0.023	7.04 (1.31–37.70)
Degree of cerebral artery stenosis					
None					1.00 (Reference)
Mild	−3.33	1.19	−2.79	0.005	0.04 (0.00–0.37)
Moderate or severe	−0.10	0.33	−0.30	0.764	0.90 (0.47–1.74)
Occlusion	0.21	0.40	0.52	0.605	1.23 (0.56–2.67)
Low density lipoprotein	−0.37	0.14	−2.53	0.011	0.69 (0.52–0.92)
Neutrophil	0.06	0.01	4.40	<0.001	1.07 (1.04–1.10)
Education level					
Illiterate					1.00 (Reference)
Primary school	−4.62	1.50	−3.08	0.002	0.01 (0.00–0.19)
Secondary school	−5.02	1.50	−3.35	<0.001	0.01 (0.00–0.12)
College or above	−4.14	1.57	−2.63	0.008	0.02 (0.00–0.35)
C-reactive protein	0.02	0.01	3.13	0.002	1.02 (1.01–1.03)
Number of infarcts	0.78	0.14	5.54	<0.001	2.17 (1.65–2.86)

OR, odds ratio; CI: confidence interval.

The multivariable logistic regression analysis identified several factors independently associated with an increased risk of post-stroke cognitive impairment (PSCI). The strongest anatomical risk factor was infarction in the corpus callosum (OR = 7.04, 95% CI: 1.31–37.70), suggesting that lesions disrupting this key interhemispheric connectivity hub confer a substantially higher risk. Higher educational attainment was a strong, graded protective factor; all educated groups showed markedly reduced odds of PSCI compared to the illiterate reference group (all ORs ≤0.02). Among continuous variables, a higher number of cerebral infarcts (OR = 2.17 per infarct, 95% CI: 1.65–2.86) and elevated neutrophil count (OR = 1.07 per unit, 95% CI: 1.04–1.10) were significantly associated with increased risk, potentially reflecting increased inflammatory or overall disease burden. Conversely, higher low-density lipoprotein levels were associated with a lower risk in this cohort (OR = 0.69, 95% CI: 0.52–0.92), a finding that requires careful interpretation and contextualization within the existing literature.

### 3.6 Nomogram of cognitive impairment patients after acute ischemic stroke

**Figure 5:**
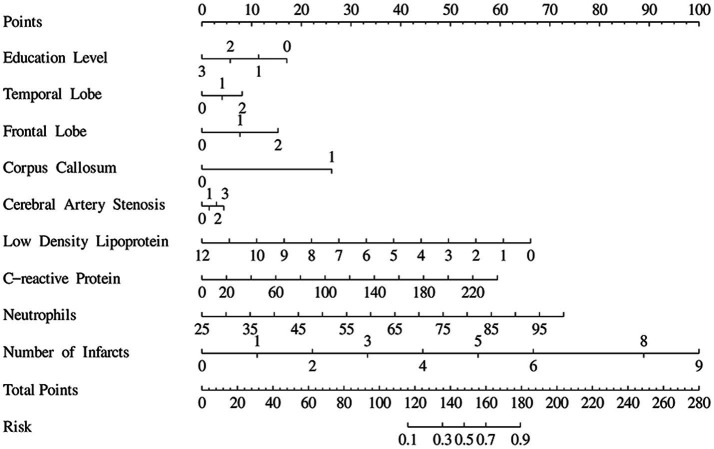
Nomogram for patients with cognitive impairment after acute ischemic stroke.

A nomogram was constructed based on a logistic regression model to predict the risk of an event. Each variable in the figure corresponded to a horizontal scale, and the corresponding “Points” (score) could be determined according to the value of the variable. The “Total Points” were calculated by adding up the scores for each variable. The total score corresponded to the “Risk” scale at the bottom, which estimated the probability of the event. For example, the value of “Low density lipoprotein” ranged from 0 to 12, and if the value was 6, the corresponding score was approximately 33. By analogy, the corresponding “Points” were also calculated for other variables, and finally the total score was obtained and the risk was estimated. The purpose of this figure was to visually show the impact of each variable on the predicted results and to facilitate individual risk assessment (Figure 5).

## 4 Discussion

In this study, we developed a validated predictive model for cognitive impairment after first-episode AIS, which demonstrated excellent discriminatory performance in both internal and external cohorts. The key predictors identified and integrated into the model included number of infarcts, frontal lobe infarction, the levels of biomarkers and low education level, among others, which are closely related to the decline in cognitive function. These findings are consistent with existing literature (17, 18), and provide empirical support for the importance of early identification of high-risk patients. This study demonstrated that the incidence of cognitive impairment at 6 months after first-episode AIS was 29.98%, this is consistent with the incidence rate of cognitive impairment after AIS reported in previous studies (4, 5).

The results of this study showed that an increased number of infarcts was linked to a higher risk of cognitive impairment in patients. This result is consistent with multiple previous studies, such as the study by Hou et al. (19) in China, which found that patients with higher number of infarcts have relatively poor cognitive function recovery and a significantly increased risk of developing dementia in the later stages. Especially when the infarction location involves the frontal and temporal lobes, cognitive impairment is more pronounced. Frontal lobe injury is usually associated with executive dysfunction (20), while temporal lobe injury has a serious impact on memory and language abilities (21). This has been fully validated in our results, emphasizing the importance of early identification of these critical area infarcts in order to take targeted treatment measures.

It has been found that the elevation of inflammatory markers such as C-reactive protein (CRP) is closely related to the occurrence of cognitive impairment after AIS (22). These biomarkers can not only reflect the systemic inflammatory status of patients, but also provide useful information for predicting changes in cognitive function. Many related studies have shown that the increase of CRP level is directly proportional to the risk of cognitive impairment (23). This suggests its potential value for clinical application.

This study has also demonstrated that higher education level is an independent protective factor against cognitive impairment after AIS, a conclusion that aligns with multiple prior reports and is coherently interpreted through the “cognitive reserve” theory (24). Extended engagement in formal education is regarded as a principal contributor to the development of cognitive reserve. During early life, individuals with more years of education receive sustained and structured cognitive training, which fosters synaptic density and complexity, improves the efficiency and flexibility of neural network architecture, and is associated with increased gray matter volume in key regions, including the prefrontal cortex (25). Thus, in the context of AIS-induced damage to localized brain functions, the more robust cognitive reserve observed among highly educated patients may enable effective compensation for the cognitive deficits directly resulting from cerebral lesions.

This study did not observe that basic diseases (such as hypertension and diabetes) were significantly associated with cognitive impairment after acute ischemic stroke, although chronic diseases such as hypertension and diabetes were confirmed in some studies to significantly increase the risk of cognitive impairment in patients. Hughes et al. (26) and Zheng et al. (27) found that controlling blood pressure can reduce the risk of cognitive impairment. Murich et al. (28) showed that hyperglycemia in diabetes patients may also accelerate brain damage through oxidative stress and inflammatory reaction. This discrepancy may be attributed to differences in the study populations. The above two studies were included in the community health population or non-ischemic stroke patients, while our study included patients with acute ischemic stroke. Previous studies showed that the prevalence of hypertension and diabetes in patients with acute ischemic stroke was significantly higher than that in healthy people.

The machine learning model in this study adopts model interpretability technology, which, through specific techniques, makes the decision-making process and outputs of a complex model understandable. This technology has been less applied in previous research. With the development of artificial intelligence technology, the interpretability of models has become increasingly important, as it can not only improve the transparency and credibility of models, but also help users better understand and apply models (29, 30).

Although this study offers valuable insights into cognitive impairment after AIS, several limitations should be acknowledged. First, participants were predominantly recruited from a single geographic area, potentially introducing selection bias and limiting the generalizability of the findings. Future research should consider multicenter designs with expanded cohorts to improve the external validity of the model. Second, the potential influence of rehabilitation interventions on cognitive recovery was not systematically evaluated. Third, several variables that may contribute to cognitive outcomes—including socioeconomic status and occupational history—were not fully accounted for in the present analysis.

The occurrence of cognitive impairment after acute ischemic stroke is a complex process involving multiple biological mechanisms, psychological factors, and social support. Future research should focus on the development of early detection technologies and personalized treatment plans to address this increasingly severe public health challenge.

## 5 Conclusion

In this study, we successfully constructed a predictive model for cognitive impairment after first-episode AIS based on multifactor analysis and machine learning, which demonstrated excellent performance in external validation. The results showed that the number of infarcts, frontal lobe, and cerebral artery stenosis were important factors in predicting cognitive impairment after AIS. Future research should consider larger scale multicenter prospective studies to further validate the stability and applicability of the model. In addition, more potential biomarkers and intervention measures should be explored to improve the accuracy and wide applicability of predictive models.

The high discriminatory accuracy of our model, coupled with the user-friendly nomogram, translates into direct practical utility for early risk assessment. It provides clinicians with a quantifiable means to stratify patients at highest risk during the acute phase, thereby facilitating timely referral for comprehensive cognitive evaluation and potential early intervention.

## Data Availability

The original contributions presented in the study are included in the article/Supplementary material, further inquiries can be directed to the corresponding author.
