# Supplementary material for: Development and validation of a predictive model for cognitive impairment after first-episode acute ischemic stroke without reperfusion therapy
Source: Front Neurol. 2026 Mar 6;17:1731060. doi: 10.3389/fneur.2026.1731060 (PMC13002453; doi:10.3389/fneur.2026.1731060)
Supplement: Supplementary file 1 [file Supplementary_file_1.docx]

Supplementary Material

# Supplementary Figures and Tables

## Supplementary Figures

**
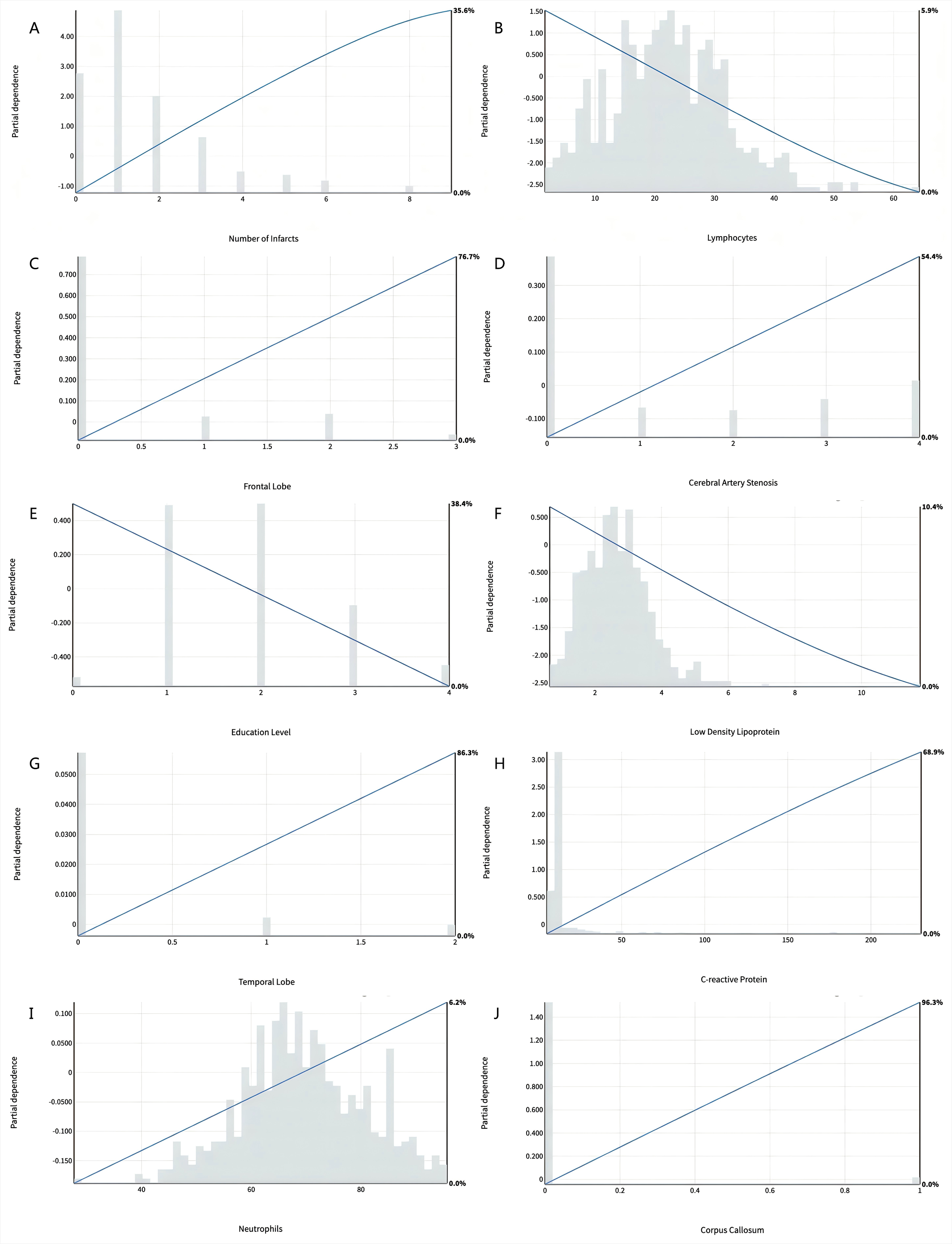
**

**Supplementary Figure 1.** Effect of different factors on the risk of occurrence cognitive impairment after acute ischemic stroke

The abscissa in the figure is the change of the value of each variable, and the left y-axis > 0 indicates an increased risk of cognitive impairment, while < 0 indicates a decreased risk., and the right ordinate is the probability of risk occurrence.

## Supplementary Tables

**Supplementary Table 1.** Quantitative variables

| Variables  Mean ± SD | Total cohort  (n = 627) | Non-impaired group  (n = 439) | Cognitively impaired group  (n = 188) | Test value | P value |
| --- | --- | --- | --- | --- | --- |
|  |  |  |  |  |  |
| Smoking history  (years) | 5.97 ± 13.11 | 7.44 ± 14.21 | 2.54 ± 9.25 | t=5.12 | <.001 |
| Alcohol drinking  (years) | 5.33 ± 12.77 | 6.23 ± 13.29 | 3.24 ± 11.22 | t=2.88 | 0.004 |
| Blood glucose | 6.75 ± 3.05 | 6.69 ± 2.88 | 6.88 ± 3.42 | t=-0.70 | 0.486 |
| Total Cholesterol | 4.34 ± 1.18 | 4.52 ± 1.18 | 3.93 ± 1.08 | t=5.88 | <.001 |
| Triglycerides | 1.65 ± 1.20 | 1.75 ± 1.33 | 1.40 ± 0.75 | t=4.22 | <.001 |
| Triglyceride Glucose Index | 1.49 ± 0.69 | 1.54 ± 0.72 | 1.39 ± 0.60 | t=2.64 | 0.009 |
| High density Lipoprotein (HDL) | 1.10 ± 0.31 | 1.13 ± 0.30 | 1.04 ± 0.32 | t=3.39 | <.001 |
| Low density Lipoprotein (LDL) | 2.67 ± 1.03 | 2.82 ± 1.03 | 2.32 ± 0.97 | t=5.65 | <.001 |
| Neutrophil | 69.40 ± 11.32 | 66.46 ± 10.47 | 76.28 ± 10.19 | t=-10.85 | <.001 |
| Lymphocyte | 22.12 ± 9.80 | 24.69 ± 9.35 | 16.12 ± 8.05 | t=10.95 | <.001 |
| Neutrophils Lymphocyte ratio | 4.72 ± 5.14 | 3.63 ± 3.51 | 7.27 ± 7.09 | t=-6.70 | <.001 |
| C-reactive protein | 16.31 ± 25.64 | 11.39 ± 14.72 | 27.79 ± 38.78 | t=-5.63 | <.001 |
| Age | 66.63 ± 11.76 | 65.30 ± 11.33 | 69.75 ± 12.16 | t=-4.41 | <.001 |
| Number of infarcts | 1.66 ± 1.64 | 1.05 ± 1.04 | 3.09 ± 1.88 | t=-13.97 | <.001 |

# Supplementary Table 2. Qualitative variables

| Variables n (%) | Total cohort  (n = 627) | Non-impaired group  (n = 439) | Cognitively impaired group  (n = 188) | Test value | P value |
| --- | --- | --- | --- | --- | --- |
|  |  |  |  |  |  |
| Gender |  |  |  | χ²=0.06 | 0.807 |
| Female | 219 (34.93) | 152 (34.62) | 67 (35.64) |  |  |
| Male | 408 (65.07) | 287 (65.38) | 121 (64.36) |  |  |
| Marital status |  |  |  | χ²=1.72 | 0.638 |
| Unmarried | 10 (1.59) | 7 (1.59) | 3 (1.60) |  |  |
| Married | 613 (97.77) | 428 (97.49) | 185 (98.40) |  |  |
| Other | 4 (0.64) | 4 (0.91) | 0 (0.00) |  |  |
| Education level |  |  |  | χ²=37.89 | <.001 |
| Illiterate | 12 (1.91) | 1 (0.23) | 11 (5.85) |  |  |
| Primary School | 239 (38.12) | 151 (34.40) | 88 (46.81) |  |  |
| Secondary School | 348 (55.51) | 266 (60.59) | 82 (43.61) |  |  |
| College or Above | 28 (4.47) | 21 (4.79) | 7 (3.72) |  |  |
| Hypertensive |  |  |  | χ²=4.46 | 0.216 |
| Not have | 123 (19.62) | 87 (19.82) | 36 (19.15) |  |  |
| Level 1 | 10 (1.59) | 10 (2.28) | 0 (0.00) |  |  |
| Level 2 | 39 (6.22) | 27 (6.15) | 12 (6.38) |  |  |
| Level 3 | 455 (72.57) | 315 (71.75) | 140 (74.47) |  |  |
| Diabetes |  |  |  | χ²=2.17 | 0.303 |
| Not have | 375 (59.81) | 266 (60.59) | 109 (57.98) |  |  |
| Type 1 | 3 (0.48) | 1 (0.23) | 2 (1.06) |  |  |
| Type 2 | 249 (39.71) | 172 (39.18) | 77 (40.96) |  |  |
| Coronary heart Disease |  |  |  | χ²=17.13 | <.001 |
| Not have | 528 (84.21) | 387 (88.15) | 141 (75.00) |  |  |
| There are | 99 (15.79) | 52 (11.85) | 47 (25.00) |  |  |
| Frontal lobe infarction |  |  |  | χ²=154.48 | <.001 |
| Not have | 481 (76.71) | 396 (90.21) | 85 (45.21) |  |  |
| Left side | 77 (12.29) | 18 (4.10) | 59 (31.39) |  |  |
| Right side | 69 (11.00) | 25 (5.69) | 44 (23.40) |  |  |
| Temporal lobe infarction |  |  |  | χ²=116.98 | <.001 |
| Not have | 541 (86.28) | 421 (95.90) | 120 (63.83) |  |  |
| Left side | 54 (8.61) | 8 (1.82) | 46 (24.47) |  |  |
| Right side | 32 (5.10) | 10 (2.28) | 22 (11.70) |  |  |
| Parietal infarction |  |  |  | χ²=108.94 | <.001 |
| Not have | 511 (81.50) | 403 (91.80) | 108 (57.45) |  |  |
| Left side | 58 (9.25) | 12 (2.73) | 46 (24.47) |  |  |
| Right side | 46 (7.34) | 19 (4.33) | 27 (14.36) |  |  |
| Bilateral | 12 (1.91) | 5 (1.14) | 7 (3.72) |  |  |
| Occipital lobe infarction |  |  |  | χ²=55.07 | <.001 |
| Not have | 541 (86.28) | 406 (92.48) | 135 (71.81) |  |  |
| Left side | 46 (7.34) | 12 (2.73) | 34 (18.09) |  |  |
| Right side | 35 (5.58) | 19 (4.33) | 16 (8.51) |  |  |
| Bilateral | 5 (0.80) | 2 (0.46) | 3 (1.60) |  |  |
| Insular leaf infarction |  |  |  | χ²=36.33 | <.001 |
| Not have | 595 (94.90) | 431 (98.18) | 164 (87.23) |  |  |
| Left side | 14 (2.23) | 1 (0.23) | 13 (6.91) |  |  |
| Right side | 18 (2.87) | 7 (1.59) | 11 (5.85) |  |  |
| Thalamic infarction |  |  |  | χ²=7.36 | 0.078 |
| Not have | 585 (93.30) | 413 (94.08) | 172 (91.49) |  |  |
| Left side | 16 (2.55) | 11 (2.51) | 5 (2.66) |  |  |
| Right side | 23 (3.67) | 15 (3.42) | 8 (4.26) |  |  |
| Bilateral | 3 (0.48) | 0 (0.00) | 3 (1.60) |  |  |
| Cerebellar infarction |  |  |  | χ²=8.14 | 0.054 |
| Not have | 590 (94.10) | 417 (94.99) | 173 (92.02) |  |  |
| Left side | 15 (2.39) | 11 (2.51) | 4 (2.13) |  |  |
| Right side | 14 (2.23) | 9 (2.05) | 5 (2.66) |  |  |
| Bilateral | 8 (1.28) | 2 (0.46) | 6 (3.19) |  |  |
| Brainstem infarction |  |  |  | χ²=0.03 | 0.857 |
| Not have | 548 (87.40) | 383 (87.24) | 165 (87.77) |  |  |
| There are | 79 (12.60) | 56 (12.76) | 23 (12.23) |  |  |
| Corpus callosum |  |  |  | χ²=47.06 | <.001 |
| Not have | 604 (96.33) | 437 (99.54) | 167 (88.83) |  |  |
| There are | 23 (3.67) | 2 (0.46) | 21 (11.17) |  |  |
| Basal ganglia infarction |  |  |  | χ²=34.30 | <.001 |
| Not have | 380 (60.61) | 286 (65.15) | 94 (50.00) |  |  |
| Left side | 80 (12.76) | 56 (12.76) | 24 (12.77) |  |  |
| Right side | 65 (10.37) | 50 (11.39) | 15 (7.98) |  |  |
| Bilateral | 102 (16.27) | 47 (10.71) | 55 (29.26) |  |  |
| Subcortical infarction |  |  |  | χ²=0.18 | 0.668 |
| Not have | 399 (63.64) | 277 (63.10) | 122 (64.89) |  |  |
| There are | 228 (36.36) | 162 (36.90) | 66 (35.11) |  |  |
| White matter hyperintensities |  |  |  | χ²=7.44 | 0.006 |
| Not have | 413 (65.87) | 304 (69.25) | 109 (57.98) |  |  |
| There are | 214 (34.13) | 135 (30.75) | 79 (42.02) |  |  |
| Cerebral artery stenosis |  |  |  | χ²=80.63 | <.001 |
| Not have | 341 (54.39) | 261 (59.45) | 80 (42.55) |  |  |
| Mild | 56 (8.93) | 55 (12.53) | 1 (0.53) |  |  |
| Moderate or Severe | 123 (19.61) | 82 (18.68) | 41 (21.81) |  |  |
| Occlusion | 107 (17.07) | 41 (9.34) | 66 (35.11) |  |  |
| Arterial stenosis |  |  |  | χ²=26.78 | <.001 |
| Not have | 375 (59.81) | 259 (59.00) | 116 (61.70) |  |  |
| Mild | 131 (20.89) | 97 (22.10) | 34 (18.09) |  |  |
| Moderate | 56 (8.93) | 41 (9.34) | 15 (7.98) |  |  |
| Severe | 50 (7.97) | 40 (9.11) | 10 (5.32) |  |  |
| Occlusion | 15 (2.39) | 2 (0.46) | 13 (6.91) |  |  |
| vertebral artery stenosis |  |  |  | χ²=2.25 | 0.863 |
| Not have | 592 (94.42) | 412 (93.85) | 180 (95.74) |  |  |
| Mild | 5 (0.80) | 4 (0.91) | 1 (0.53) |  |  |
| Moderate | 5 (0.80) | 4 (0.91) | 1 (0.53) |  |  |
| Severe | 4 (0.64) | 4 (0.91) | 0 (0.00) |  |  |
| Occlusion | 21 (3.35) | 15 (3.42) | 6 (3.19) |  |  |
| χ²: Chi-square test, -: Fisher exact | | | | | |

**Supplementary Table 3.** hyperparameter tuning

1.Logistic Regression

Logistic regression was selected as the final model.

Despite its name, Logistic Regression is a classification algorithm, using a linear model (i.e., it computes the target feature as a linear combination of input features).

Logistic Regression minimizes a specific cost function (called logit or sigmoid function), which makes it appropriate for classification.

A simple Logistic regression algorithm is prone to overfitting and sensitive to errors in the input dataset. To address these issues, it is possible to use a penalty (or regularization term) to the weights.

Regularization can be applied using either L1 or L2 penalty.

The settings for this algorithm are given below. For hyperparameters, the possible values or ranges are listed:

| Hyperparameter | Tested Values / Range |
| --- | --- |
| Regularization | Try with L1 regularization: Yes Try with L2 regularization: No |
| C | 0.01 0.1 1 10 100 |

2.Random Forest

A Random Forest is made of many decision trees. Each tree in the forest predicts a record, and each tree "votes" for the final answer of the forest.

The forest chooses the class having the most votes.

A decision tree is a simple algorithm which builds a decision tree. Each node of the decision tree includes a condition on one of the input features.

When "growing" (ie, training) the forest:

- for each tree, a random sample of the training set is used;

- for each decision point in the tree, a random subset of the input features is considered.

Random Forests generally provide good results, at the expense of "explainability" of the model.

| Hyperparameter | Tested Values / Range |
| --- | --- |
| Number of trees | 100 |
| Feature sampling strategy | Square root |
| Number of features to sample | 5 |
| Proportion of features to sample | 0.3 |
| Maximum depth of tree | 8 |
| Minimum samples per leaf | 1 |
| Number of threads for parallel processing | 4 |
| Allow sparse matrices | Yes |

3.Gradient Boosted Trees

Gradient boosting is a technique which produces a prediction model in the form of an ensemble of "weak" prediction models (small decision trees).

The concept is to train a set of decision trees (weak learners) to create a final strong learner. This is an iterative method. After each tree is trained, the data is reweighted: samples that were misclassified gain weight while the correctly classified ones lose weight. This allows future weak learners to focus on the "difficult" examples that the previous weak learners missed.

Gradient Boosted Trees is a generalization of boosting to arbitrary differentiable loss functions. GBT is an accurate and effective off-the-shelf procedure that can be used for both regression and classification problems. Gradient Boosted Trees models are used in a variety of areas including Web search ranking and ecology. The advantages of GBRT are:

- Natural handling of data of mixed type (= heterogeneous features)

- Predictive power

- Robustness to outliers in output space (via robust loss functions)

Due to the iterative nature of boosting, it is not very parallelizable and is less scalable than other algorithms.

| Hyperparameter | Tested Values / Range |
| --- | --- |
| Number of boosting stages | 100 |
| Feature sampling strategy | Square root |
| Number of features to sample | 0 |
| Proportion of features to sample | 0.3 |
| Learning rate | 0.1 |
| Loss | Try Deviance: Yes Try Exponential: No |
| Maximum depth of tree | 3 |
| Minimum samples per leaf | 1 |
| Allow sparse matrices | Yes |

4.LightGBM

LightGBM is a tree-based gradient boosting library designed to be distributed and efficient. This algorithm provides fast training speed, low memory usage, good accuracy and is capable of handling large scale data.

For more information on gradient tree boosting, see the "Gradient tree boosting" algorithm.

| Hyperparameter | Tested Values / Range |
| --- | --- |
| Boosting type | Try Gradient Boosting Decision Tree: Yes Try Gradient One-Side sampling: No |
| Maximum number of trees | 75 |
| Maximum depth of trees | -1 |
| Number of leaves | 31 |
| Learning rate | 0.1 0.2 |
| Minimum split gain | 0 |
| Minimum child weight | 0.001 |
| Minimum leaf samples | 10 30 |
| Columns subsample ratio for trees | 0.7 0.9 |
| L1 regularization | 0 |
| L2 regularization | 0 |
| Use bagging | No |
| Early stopping | Yes |
| Early stopping rounds | 4 |
| Random state | 1337 |
| Parallelism | 4 |
| Allow sparse matrices | Yes |

5.XGBoost

XGBoost is an advanced gradient tree boosting algorithm. It has support for parallel processing, regularization and early stopping, which makes it a fast, scalable and accurate algorithm.

For more information on gradient tree boosting, see the "Gradient tree boosting" algorithm.

| Hyperparameter | Tested Values / Range |
| --- | --- |
| Booster | Try Gradient Boosted Trees: Yes Try DART: No |
| Tree method | Automatic - CPU only |
| Maximum number of trees | 300 |
| Early stopping | Yes |
| Early stopping rounds | 4 |
| Maximum depth of tree | 3 |
| Learning rate | 0.2 |
| Max delta step | 0 |
| L2 regularization | 1 |
| L1 regularization | 0 |
| Gamma | 0 |
| Minimum child weight | 1 |
| Subsample | 1 |
| Columns subsample ratio for trees | 1 |
| Columns subsample ratio for splits / levels | 1 |
| Parallelism | 4 |
| Allow sparse matrices | Yes |
| Custom missing value | No |

6.Decision Tree

Decision Tree is a simple non-parametric algorithm. It creates a model that predicts the value of the target by learning simple decision rules inferred from the data features.

These rules form a tree, with the leaves of the tree carrying the predicted class. Evaluation simply goes down the tree and evaluates the rule at each split.

| Hyperparameter | Tested Values / Range |
| --- | --- |
| Maximum depth | 5 |
| Criterion | Try Gini: Yes Try Entropy: No |
| Min. samples per leaf | 1 |
| Split strategy | Try Best: Yes Try Random: No |

7.Support Vector Machine

Support Vector Machine is a powerful (but very slow) 'black-box' algorithm for classification.

Through the use of kernel functions, it can learn complex non-linear decision boundaries (i.e., when it is not possible to compute the target as a linear combination of input features).

SVM is effective with a large number of features. However, this algorithm is generally much slower than others and is generally not practical with more than a few thousand records.

| Hyperparameter | Tested Values / Range |
| --- | --- |
| Kernel | Try with a linear kernel: No Try with a RBF kernel: Yes Try with a polynomial kernel: No Try with a sigmoid kernel: No |
| C | 1 |
| Gamma | Try with 1/nb_features (auto): No Try with 1/(nb_features * variance) (scale): Yes Try with custom gamma values: No |
| Custom Gamma values | 0.001 |
| Tolerance | 0.001 |
| Maximum number of iterations | -1 |

**Supplementary Table 4.** multicollinearity diagnostics

|  | | |
| --- | --- | --- |
| Variables | VIF | Tolerance |
| Low Density Lipoprotein | 1.030 | 0.970 |
| Neutrophils | 8.404 | 0.119 |
| Lymphocytes | 8.233 | 0.121 |
| C-reactive Protein | 1.202 | 0.832 |
| Education Level | 1.088 | 0.919 |
| Frontal Lobe | 1.474 | 0.678 |
| Temporal Lobe | 1.543 | 0.648 |
| Corpus Callosum | 1.250 | 0.800 |
| Number of Infarcts | 1.892 | 0.529 |
| Cerebral Artery Stenosis | 1.235 | 0.809 |

Results of the multicollinearity analysis using Variance Inflation Factor (VIF) are presented in the table. VIF values > 5 indicated multicollinearity between Neutrophils and Lymphocytes. Lymphocytes were excluded from the final model to avoid multicollinearity.
